# Determine the Optimal Extent of Thyroidectomy and Lymphadenectomy for Patients With Papillary Thyroid Microcarcinoma

**DOI:** 10.3389/fendo.2019.00363

**Published:** 2019-06-19

**Authors:** Jia-Wei Feng, Hua Pan, Lei Wang, Jing Ye, Yong Jiang, Zhen Qu

**Affiliations:** The Third Affiliated Hospital of Soochow University, Changzhou First People's Hospital, Changzhou, China

**Keywords:** papillary thyroid microcarcinoma, central lymph node metastasis, central lymph node dissection, recurrence-free survival, surgery

## Abstract

**Background:** The optimal extent of surgery, including lymph node dissection, remains controversial in papillary thyroid microcarcinoma (PTMC). Determining risk factors of central lymph node metastasis (CLNM) and recurrence-free survival (RFS) may help surgeons determine individualized surgery.

**Methods:** A total of 353 patients with PTMC were retrospectively analyzed, including 263 with overt PTMC and 90 with incidental PTMC. The recurrence rates between different extents of thyroidectomy were compared. The relationship between CLNM and clinicopathologic factors was analyzed. The Cox regression model was used to determine the risk factors for RFS.

**Results:** Lobectomy/total thyroidectomy (TT) with central neck dissection (CND) was performed in 263 overt PTMC patients, and lobectomy/partial thyroidectomy was performed in 90 incidental PTMC patients. In 263 overt PTMC patients, 93 (26.3%) had CLNM only and 13 (3.7%) had both CLNM and lateral lymph node metastases (LLNM). Multifocal PTMC patients who underwent lobectomy had a higher rate of thyroid bed and lymph node recurrence than patients who underwent TT (*P* < 0.05). Independent predictors for CLNM were age <45 years, tumor size >5 mm and presence of extrathyroidal extension (ETE). Tumor size >5 mm, multifocality, presence of ETE, presence of CLNM, and presence of LLNM were the significant factors related to the RFS.

**Conclusion:** Fine-needle aspiration biopsy is advised to distinguish incidental PTMC from the benign nodules. For multifocal PTMC patients, TT should be performed to reduce recurrence. Routine prophylactic CND can be recommended in PTMC patients with independent risk factors of CLNM. Aggressive surgery and close follow-up are essential for patients with risk factors of RFS.

## Introduction

Thyroid cancer (TC), the most common cancer of the endocrine system, is rising at the fastest rate of all malignancies (1, 2). The most common histological form is papillary thyroid cancer (PTC), which accounts for 80–85% of TC (3–5). The World Health Organization defined papillary thyroid microcarcinoma (PTMC) as a papillary carcinoma with a maximum diameter of 10 mm or less (6). Although there are a number of clinical and imaging methods used to identify benign and malignant nodules of the thyroid gland, their practicality is limited in clinical practice. For instance, incidental PTMC is found incidentally during the removal of the thyroid for benign diseases in up to 22% among benign thyroid nodules (7). Occult PTMC presents as lymph nodes metastases (LNM) without an identifiable thyroid focus. Moreover, PTMC was reported in up to 36% on autopsy of patients who died of non-thyroid-related diseases (8).

Although the prognosis of patients with PTMC is overall excellent, LNM, especially central lymph node metastases (CLNM), is common, occurring in 12–64% of patients (9–11). LNM has been considered as a poor prognostic factor for local recurrence and survival (12, 13). Cause of the limited resolution of Ultrasonography (US), the sensitivity of US for detecting CLNM was reported to 10.9 to 30% (14, 15). The optimal treatment strategy, especially whether prophylactic central neck dissection (CND) should be performed in patients with clinically uninvolved central neck lymph nodes (cN0), has remained debated. As reported, the rate of CLNM detected intraoperatively or postoperatively by histopathological examination was up to 31–60.9% (16, 17) in cN0 PTMC patients. Prophylactic CND has the advantage of eliminating potential recurrent sources, improving the accuracy of staging and avoiding the potential incidence of reoperation (18). The American Thyroid Association (ATA) guidelines (2009) suggested that prophylactic CND in cN0 PTC patients may prevent a future recurrence, followed by the lower postoperative thyroglobulin (Tg) serum levels (19). Moreover, ATA guidelines (2015) recommended the prophylactic CND for cN0 PTC patients who have advanced primary tumors (T3 or T4) (20). However, opponents hold the view that prophylactic CND did not improve the survival and may increase the incidence of complications (21).

Therefore, determining risk factors for CLNM may help surgeons to find the optimal management of cervical lymph nodes in PTMC patients. By using a large series of overt PTMC patients who underwent lobectomy/total thyroidectomy (TT) with CND, we aimed to determine the risk factors of CLNM. By comparing the recurrence of overt PTMC patients based on the extent of thyroidectomy, we aimed to determine the optimal extent of thyroidectomy. Moreover, we investigated risk factors of Recurrence-free survival (RFS) to determine the individualized treatment.

## Materials and Methods

### Patients

This retrospective study was approved by the Institutional Review Board of Changzhou First People's Hospital. All participants gave written informed consent for their clinical records to be used in this study. Patients who underwent total thyroidectomy, lobectomy or partial thyroidectomy during the period between January 2010 and January 2018 were retrospectively reviewed from our department's prospective surgical database. The patients were excluded if they met the following criteria: (1) had another malignancy before thyroidectomy; (2) reoperation; (3) non-PTCs (medullary/follicular/anaplastic) or mixed-type PTC; (4) PTC ≥1 cm; (5) distant metastasis at diagnosis on pathological or clinical analysis; (6) history of neck radiation or familial cancer. In total, 353 patients were finally included and assessed, of which 263 were overt PTMC and 90 were incidental PTMC.

### Surgical Procedures

**Figure 1:**
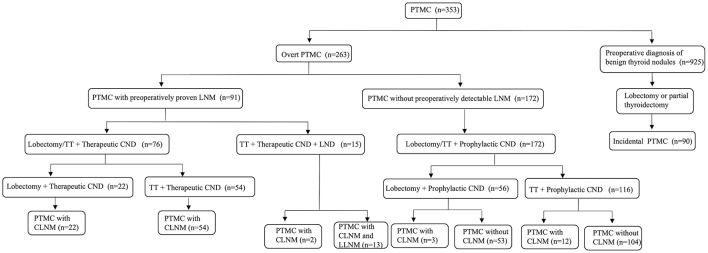
The flowchart of surgical procedures. *PTMC* papillary thyroid microcarcinoma, *LNM* lymph nodes metastases, *TT* total thyroidectomy, *CND* central neck dissection, *LND* lateral neck dissection, *CLNM* central lymph node metastasis, *LLNM* lateral lymph node metastases.

The flowchart of surgical procedures was indicated in Figure 1. PTMC was considered incidental when at least one of the following occurred: (1) the case of an incidental diagnosis at final histology for patients who underwent surgery for diseases not related to thyroid malignancy; (2) false negative fine-needle aspiration (FNA) (PTMC in a benign nodule) or unevaluated nodules. Overt PTMC was defined as the preoperatively proven or suspected malignant thyroid nodules based on FNA or other imaging diagnosis. Lobectomy was defined as the removal of the involved lobe, with or without the isthmus and the pyramidal lobe. TT was defined as the removal of two lobes, the isthmus, and the pyramidal lobe. CND included the removal of the prelaryngeal, pretracheal, and both the right and left paratracheal nodal basins (22). LND was performed in the usual fashion from at least level II to level V, sparing the internal jugular vein, spinal accessory nerve and sternocleidomastoid muscle (23). Lobectomy/TT with prophylactic CND was performed in 172 cN0 PTC patients, 15 of whom were proven to have CLNM postoperatively. Ninety-one patients were preoperatively proven or suspected to have CLNM, 15 of whom were suspected to have lateral lymph node metastases (LLNM) intraoperatively and underwent TT with therapeutic CND and ipsilateral lateral neck dissection (LND). The remaining 76 patients underwent lobectomy/TT with therapeutic CND. Postoperative pathology confirmed that among the 15 patients who underwent LND, 13 patients developed LLNM. Nine hundred and twenty-five patients were diagnosed with benign thyroid nodules and had lobectomy or partial thyroidectomy performed, 90 of whom were confirmed as having incidental PTMC postoperatively.

### Histopathologic Examination of Surgical Specimens

Two or more experienced pathologists microscopically reviewed and cross-checked all pathology specimens. Microcarcinoma was defined as PTC ≤1 cm in its maximum diameter. Multifocality was defined as two or more PTC lesions within the thyroid. Two or more PTC lesions in a single lobe were unilateral multifocal, while two or more PTC lesions in both lobes or one lobe plus isthmus were bilateral multifocal. Extrathyroidal extension (ETE) was defined as neoplastic infiltration beyond the thyroid fibrous capsule. Recurrent disease was defined as the disease with the new evidence of pathologically proven recurrence in the thyroid bed, soft tissue, cervical lymph nodes, or other organs on cytology from aspiration biopsy or reoperation. RFS was used to evaluate the outcomes. The length of time between the date of completion of the thyroid surgery and dates of recurrence was identified as the RFS.

### Postoperative Complications

Fiberoptic laryngoscopy was performed preoperatively and postoperatively in all patients to evaluate the mobility of the vocal cord. Transient recurrent laryngeal nerve (RLN) injury was regarded as decreased or absent vocal cord mobility resolving within 6 months of surgery. Impaired vocal cord mobility for more than 6 months after surgery was considered permanent RLN injury. Serum calcium and phosphorus concentrations were evaluated 1 day after surgery. Transient hypocalcemia was defined as an ionized calcium level <2.10 mmol/L during the hospital stay and the calcium level recovered normal within 6 months. Permanent hypoparathyroidism was diagnosed in patients still requiring calcium supplementation more than 6 months after surgery. Immediate emergency surgery would be performed if the postoperative incision bleeding affected breathing.

### Postoperative Management and Follow-up

Postoperative suppressive levothyroxine treatment was administered to patients. Thyroid-stimulating hormone (TSH) suppression therapy (serum TSH level below 0.5 mIU/L) with levothyroxine with or without radioactive iodine (RAI) ablation was used for patients who underwent total thyroidectomy. Physical examinations, US of the neck and serum Tg with Tg antibodies were used for all patients every 6 months for 2 years, and annually thereafter. Further imaging examinations or histological confirmation were used when the level of Tg and/or Tg antibodies was significantly elevated. Follow-up data were obtained by outpatient consultations or telephone contact.

### Statistical Analyses

All statistical analyses were carried out using SPSS v 25.0 software (Chicago, IL, USA). The continuous variables were expressed as the means ± standard deviations. The χ ^2^ test or Fisher's exact test was used, as appropriate, for categorical data. Univariate analyses for the associations between CLNM and several clinicopathologic factors of overt PTMC patients were performed by using Pearson's chi-square test or Fisher's exact test. Binary logistic regression test was used for multivariate analysis of statistically significant variables from the univariate analysis. Univariate analysis of RFS was realized including each risk factor in a Cox regression model. RFS curves were calculated using the Kaplan–Meier method, and the log-rank test was used to assess the differences between curves.

## Results

### Clinicopathological Characteristics of 353 PTMC Patients

As summarized in Table 1, there were 264 women and 89 men with a mean age of 45.9 ± 11.6 years. One hundred eighty-one patients (51.3%) were 45 years old or older and 172 (48.7%) were younger than 45. The average largest tumor diameter was 5.9 ± 2.6 mm; 177 (50.1%) were 0.5 cm or smaller and 176 (49.9%) were larger than 0.5 cm. Of 39 PTMC patients who had BRAF mutation analysis performed, 34 (96.0%) had BRAF mutation positivity. Multifocal lesions were observed in 98 patients (27.8%), of which 40 (11.3%) had unilateral multifocal nodules and 58 (16.4%) had bilateral multifocal nodules. ETE was found in 38 (10.8%) patients, and vascular invasion was found in 9 (2.5%) patients.

**Table 1:** Clinicopathological characteristics of 353 PTMC patients.

**Clinicopathological characteristics**	**Value**
Sex (M/F)	89/264
Age (Y), Mean ± SD (range)	45.9 ± 11.6
≥45, *n* (%)	181 (51.3%)
<45, *n* (%)	172 (48.7%)
Primary tumor	
Tumor size (mm), Mean±SD (range)	5.9 ± 2.6
≤ 5, *n* (%)	177 (50.1%)
>5, *n* (%)	176 (49.9%)
BRAF mutation^*^, *n* (%)	34 (96.0%)
Multifocality, *n* (%)	98 (27.8%)
ETE, *n* (%)	38 (10.8%)
Vascular invasion, *n* (%)	9 (2.5%)
LNM, *n* (%)	
CLNM only	93 (26.3%)
CLNM and LLNM	13 (3.7%)
Without LNM	157 (44.5%)
Unknown	90 (25.5%)
Surgery, *n* (%)	
Lobectomy+CND	78 (22.1%)
TT+CND	170 (48.2%)
TT+CND+LND	15 (4.2%)
Lobectomy or partial thyroidectomy	90 (25.5%)
Recurrence, *n* (%)	21 (5.9%)
LNs	12 (3.4%)
Thyroid bed	4 (1.1%)
LNs and thyroid bed	4 (1.1%)
Lung	1 (0.3%)

*PTMC, papillary thyroid microcarcinoma; M, male; F, female; Y, year; SD, standard deviation; ETE, extrathyroidal extension; LNM, lymph node metastasis; CLNM, central lymph node metastasis; LLNM, lateral lymph node metastasis; TT, total thyroidectomy; CND, central neck dissection; LND, lateral neck dissection; LN, lymph node*.

* *BRAF mutation analysis was started in 2017 and it was performed in 39 patients with PTC*.

In overt PTMC, 91 patients were detected with CLNM before surgery. Fifteen patients were suspected of LLNM during operation, and LND was performed. Two hundred and forty-eight overt PTMC patients underwent CND only. Incidental PTMC patients did not undergo prophylactic CND. The post-operative examination verified that 157 patients (44.5%) had nodes removed without metastases, 93 patients (26.3%) had CLNM only and 13 patients (3.7%) had both CLNM and LLNM.

Postoperative follow-up ranged from 9 to 89 months (average follow-up period: 39 months). During follow-up, 21 patients (5.9%) developed recurrent disease, including 12 patients (3.4%) who had cervical lymph node recurrence, 4 patients (1.1%) who had thyroid bed recurrence, 4 patients (1.1%) who had both lymph node and thyroid bed recurrence and 1 patient (0.3%) who had lung recurrence.

### Associations Between Clinicopathological Characteristics and CLNM in Overt PTMC Patients

In 263 patients with preoperative proven or suspected PTMC, factors that were associated with the presence of CLNM were sex, age, tumor size and ETE. The incidence of CLNM was higher in men (*P* = 0.037), in patients aged <45 years (*P* = 0.018), in patients with tumor size >5 mm (*P* = 0.005), and in patients with ETE (*P* < 0.001). Additionally, presence of CLNM was associated with the recurrence of cervical lymph nodes (Table 2). In multivariate analysis, age <45 years (adjusted OR: 1.815, *P* = 0.037), tumor size >5 mm (adjusted OR: 2.030, *P* = 0.016) and ETE (adjusted OR: 10.113, *P* < 0.001) were independent predictors for CLNM (Table 3).

**Table 2:** Characteristics of 263 patients with overt PTMC.

**Characteristic**	**PTMC with preoperatively proven CLNM**	**PTMC without preoperatively detectable LNM**		*P* value^**^
		**With CLNM**	**Without CLNM**	
	***n*** **=** **91 (34.6%)**	***n*** **=** **15 (5.7%)**	***n*** **=** **157 (59.7%)**	
Sex				
Male	34 (37.4%)	2 (13.3%)	35 (22.3%)	**0.037**
Female	57 (62.6%)	13 (86.7%)	122 (77.7%)	
Age (Y)				
≥45	38 (41.8%)	7 (46.7%)	90 (57.3%)	**0.018**
<45	53 (58.2%)	8 (53.3%)	67 (42.7%)	
Tumor size (mm)				
≤ 5	27 (29.7%)	7 (46.7%)	78 (49.7%)	**0.005**
>5	64 (70.3%)	8 (53.3%)	79 (50.3%)	
BRAF mutation^*^				
Absence	2 (10.5%)	0 (0.0%)	3 (15.0%)	1.000
Presence	17 (89.5%)	0 (0.0%)	17 (85.0%)	
Multifocality				
Absence	59 (64.8%)	13 (86.7%)	119 (75.8%)	0.160
Presence	32 (35.2%)	2 (13.3%)	38 (24.2%)	
ETE				
Absence	71 (78.0%)	12 (80.0%)	153 (97.5%)	**<0.001**
Presence	20 (22.0%)	3 (20.0%)	4 (2.5%)	
Vascular invasion				
Absence	87 (95.6%)	14 (93.3%)	156 (99.4%)	0.080
Presence	4 (4.4%)	1 (6.7%)	1 (0.6%)	
CLNM				
Absence	0 (0.0%)	0 (0.0%)	157 (100.0%)	–
Presence	91 (100.0%)	15 (100.0%)	0 (0.0%)	
LLNM				
Absence	78 (85.7%)	15 (100.0%)	157 (100.0%)	–
Presence	13 (14.3%)	0 (0.0%)	0 (0.0%)	
Recurrence				
LNs	4 (4.4%)	2 (13.3%)	1 (0.6%)	**0.036**
Thyroid bed	2 (2.2%)	0 (0.0%)	1 (0.6%)	0.731
Lung	1 (1.1%)	0 (0.0%)	0 (0.0%)	0.177

*PTMC, papillary thyroid microcarcinoma; Y, year; ETE, extrathyroidal extension; CLNM, central lymph node metastasis; LLNM, lateral lymph node metastasis; LN, lymph node*.

* *BRAF mutation analysis was started in 2017 and it was performed in 39 patients with PTC*.

** *P value was the result of PTMC with preoperatively proven CLNM + PTMC with postoperatively proven CLNM vs. PTMC without CLNM. The bold values mean the P <0.05*.

**Table 3:** Multivariate analysis of risk factors of CLNM in patients with overt PTMC.

**Characteristics**	**Adjusted OR**	**95% CI**	***P*-value**
Sex			
Female	1		
Male	1.561	0.831–2.934	0.166
Age (Y)			
≥45	1		
<45	1.815	1.036–3.179	**0.037**
Tumor size (mm)			
≤ 5	1		
>5	2.030	1.140–3.617	**0.016**
ETE			
Absence	1		
Presence	10.113	3.259–31.379	**<0.001**

*CLNM central lymph node metastasis, PTMC papillary thyroid microcarcinoma, OR Odds ratio, 95% CI 95% confidence interval*.

*Y, year; ETE, extrathyroidal extension. The bold values mean the P <0.05*.

Among the 111 overt PTMC patients without all three risk factors (age <45 years, tumor size >5 mm and with ETE), only 7 patients (6.3%) had CLNM, while of the 152 patients with at least one risk factor, 99 (65.1%) showed CLNM, and the difference was statistically significant (*P* < 0.001). In addition, we compared patients with only one risk factor to patients without risk factors, and we found that the rate of CLNM was significantly higher in patients with only one risk factor than in patients without risk factors (57.1 vs. 6.3%, *P* < 0.001). Compared with patients without risk factors, the rate of CLNM was also significantly higher in patients with two or three risk factors (64.3 vs. 6.3%, *P* < 0.00; 73.9 vs. 6.3%, *P* < 0.001, respectively) (Table 4). Patients with all three risk factors were at the highest risk of CLNM.

**Table 4:** Incidence of CLNM in overt PTMC patients.

	**CLNM positive (%)**	**CLNM negative (%)**	**Total**	*P*-value^*^
Total	106	157	263	
Without RFs	7 (6.3%)	104 (93.7%)	111	
With at least one RF	99 (65.1%)	53 (34.9%)	152	**<0.001**
With one RF	8 (57.1%)	6 (42.9%)	14	**<0.001**
With two RFs	74 (64.3%)	41 (35.7%)	115	**<0.001**
With three RFs	17 (73.9%)	6 (26.1%)	23	**<0.001**

*CLNM central lymph node metastasis, PTMC papillary thyroid microcarcinoma, RF risk factor*.

* *P value was the result of patients without RF vs. patients with RF. The bold values mean the P <0.05*.

### Comparison of Recurrence Based on the Extent of Thyroidectomy in Overt PTMC Patients

As is shown in Table 5, we compared the rate of recurrence in unifocal and multifocal PTMC patients based on the extent of thyroidectomy. There was no statistical difference in thyroid bed or lymph node recurrence among unifocal PTMC patients who underwent lobectomy and patients who underwent TT. However, for multifocal PTMC patients, the rate of thyroid bed recurrence was 14.3 and 0.0% in patients who underwent lobectomy and TT (*P* = 0.009), respectively. Moreover, the rate of lymph node recurrence was 35.7% in multifocal PTMC patients who underwent lobectomy, which was significantly higher than patients who underwent TT (*P* < 0.001).

**Table 5:** Comparison of recurrence based on the extent of thyroidectomy in overt PTMC patients.

**Recurrence**	**Lobectomy**	**TT**	***P-*value**
Unifocal	64	127	
Thyroid bed	1 (1.6%)	0 (0.0%)	0.138
LNs	1 (1.6%)	0 (0.0%)	0.138
Lung	0 (0.0%)	0 (0.0%)	–
Multifocal	14	58	
Thyroid bed	2 (14.3%)	0 (0.0%)	**0.009**
LNs	5 (35.7%)	1 (1.7%)	**<0.001**
Lung	1 (7.1%)	0 (0.0%)	0.068

*TT, Total thyroidectomy; LN, lymph node. The bold values mean the P <0.05*.

### Comparison of Clinicopathological Characteristics Between Incidental PTMC and Overt PTMC

**Figure 2:**
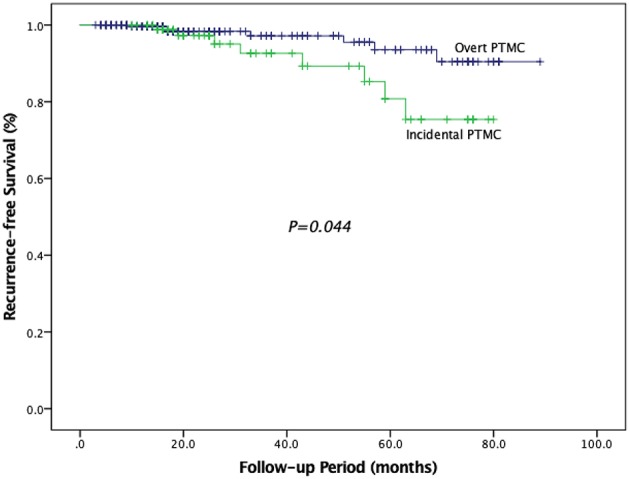
Recurrence–free survival in patients with overt PTMC and incidental PTMC.

Differences of clinicopathological characteristics between incidental PTMC and overt PTMC are compared in Table 6. The number of patients with tumor size >5 mm was less in the incidental group than in the overt group (27.8 vs. 57.4%, *P* < 0.001). In terms of postoperative recurrence, 11 (12.2%) of the 90 incidental PTMC patients who underwent lobectomy or partial thyroidectomy only developed recurrence, and 9 of these had lymph node recurrence while 5 of these had residual thyroid bed recurrence. Among 263 overt PTMC patients, 10 (3.8%) patients who underwent lobectomy/TT with CND or with CND and LND developed recurrence, and 7 of these had lymph node recurrence, three of these had residual thyroid bed recurrence and one of these had lung recurrence. Overall recurrence, cervical lymph node recurrence and thyroid bed recurrence were significantly different between incidental PTMC and overt PTMC (12.2 vs. 3.8%, *P* = 0.004; 10.0 vs. 2.7%, *P* = 0.009; 5.6 vs. 1.1%, *P* = 0.044; respectively). In addition, RFS was compared between two groups, and patients with incidental PTMC had a significantly lower RFS than patients with overt PTMC (*P* = 0.044) (Figure 2).

**Table 6:** Comparison of clinicopathological characteristics between incidental and overt PTMC.

**Characteristic**	**Incidental PTMC**	**Overt PTMC**	***P*-value**
	***n* = 90 (25.5%)**	***n* = 263 (74.5%)**	
Sex			
Male	18 (20.0%)	71 (27.0%)	0.187
Female	72 (80.0%)	192 (73.0%)	
Age (Y)			
≥45	46 (51.1%)	135 (51.3%)	0.971
<45	44 (48.9%)	128 (48.7%)	
Tumor size (mm)			
≤ 5	65 (72.2%)	112 (42.6%)	**<0.001**
>5	25 (27.8%)	151 (57.4%)	
BRAF mutation^*^			
Absence	0 (0.0%)	5 (12.8%)	–
Presence	0 (0.0%)	34 (87.2%)	
Multifocality			
Absence	64 (71.1%)	191 (72.6%)	0.782
Presence	26 (28.9%)	72 (27.4%)	
ETE			
Absence	79 (87.8%)	236 (89.7%)	0.605
Presence	11 (12.2%)	27 (10.3%)	
Vascular invasion			
Absence	87 (96.7%)	257 (97.7%)	0.874
Presence	3 (3.3%)	6 (2.3%)	
Surgery			
Lobectomy+CND	0 (0.0%)	78 (29.7%)	**<0.001**
TT+CND	0 (0.0%)	170 (64.6%)	
TT+CND+LND	0 (0.0%)	15 (5.7%)	
Lobectomy or partial thyroidectomy	90 (100.0%)	0 (0.0%)	
Recurrence^**^			
Overall	11 (12.2%)	10 (3.8%)	**0.004**
LNs	9 (10.0%)	7 (2.7%)	**0.009**
Thyroid bed	5 (5.6%)	3 (1.1%)	**0.044**
Lung	0 (0.0%)	1 (0.3%)	0.443

*PTMC papillary thyroid microcarcinoma; Y, year; ETE, extrathyroidal extension; TT, total thyroidectomy; CND, central neck dissection; LND, lateral neck dissection; LN, lymph node*.

* *BRAF mutation analysis was started in 2017 and it was performed in 39 patients with PTC*.

** *More than one recurrence occurred in some patients. The bold values mean the P <0.05*.

### Postoperative Complications

As is shown in Table 7, the incidence of postoperative complications between different groups was summarized. Fourteen (15.6%) of 90 patients with incidental PTMC developed postoperative complications, which was significantly less than patients (30.8%) with overt PTMC (*P* = 0.005). The incidence of incision infection and bleeding requiring resurgery was similar between two groups. The rates of transient RLN injury and permanent RLN injury were 2.2 and 1.1% in incidental PTMC patients, and were 3.0 and 1.5% in overt PTMC patients, and there was no statistical difference between the two groups (*P* = 0.971, *P* = 1.00, respectively). Transient hypocalcemia occurred in 62 (23.6%) overt PTMC patients, which was significantly higher than the 10 (11.1%) incidental PTMC patients (*P* = 0.011). Similarly, overt PTMC patients had a higher rate of permanent hypocalcemia than incidental PTMC patients (1.1 vs. 7.2%, *P* = 0.030). There were no postoperative deaths.

**Table 7:** Comparison of postoperative complications between incidental and overt PTMC.

**Postoperative complications**	**Incidental PTMC**	**Overt PTMC**	***P*-value**
	***n* = 90 (25.5%)**	***n* = 263 (74.5%)**	
Overall complications^*^	14 (15.6%)	81 (30.8%)	**0.005**
RLN injury			
Transient	2 (2.2%)	8 (3.0%)	0.971
Permanent	1 (1.1%)	4 (1.5%)	1.000
Hypocalcemia			
Transient	10 (11.1%)	62 (23.6%)	**0.011**
Permanent	1 (1.1%)	19 (7.2%)	**0.030**
Incision infection	0 (0.0%)	4 (1.5%)	0.549
Bleeding required resurgery	2 (2.2%)	10 (3.8%)	0.706

*PTMC papillary thyroid microcarcinoma, RLN recurrent laryngeal nerve*.

* *More than one complication occurred in some patients. The bold values mean the P <0.05*.

### Predictors of RFS

**Figure 3:**
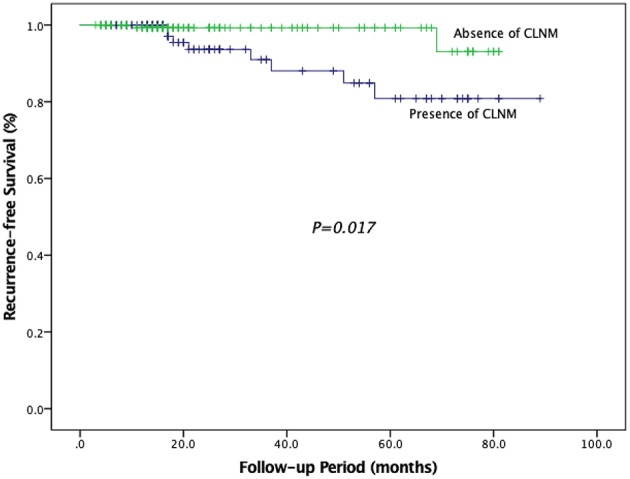
Recurrence–free survival in patients with CLNM and patients without CLNM.

We conducted univariate analyses in relation to RFS to determine the single variable which influenced risk of recurrence (Table 8). Our results showed that tumor size >5 mm, multifocality, presence of ETE, presence of CLNM, and presence of LLNM were the significant factors related to RFS (*P* = 0.047, *P* = 0.003, *P* < 0.001, *P* = 0.014, *P* < 0.001, respectively), while other investigated variables had no influence on RFS. Patients with tumor size >5 mm had a risk of recurrence 3.517 times that of patients with tumor size ≤ 5 mm. Patients with ETE had a risk of recurrence 8.831 times higher than that of patients without ETE. There was a risk of recurrence that was 10.440 times higher in patients with multifocality. Similarly, risk of recurrence was 4.624 and 20.243 times higher among patients with CLNM and LLNM, respectively. Moreover, the RFS in patients with CLNM was significantly lower than that in patients without CLNM (*P* = 0.017) (Figure 3).

**Table 8:** Cox proportional hazards model demonstrating factors associated with recurrence-free survival in overt PTMC patients.

**Characteristics**	**HR**	**95% CI**	***P-*value**
Sex			
Female	1		
Male	1.665	0.442–6.267	0.451
Age (Y)			
≥45	1		
<45	2.676	0.974–7.348	0.056
Tumor size (mm)			
≤ 5	1		
>5	3.517	1.019–12.140	**0.047**
Multifocality			
Absence	1		
Presence	10.440	2.199–49.563	**0.003**
ETE			
Absence	1		
Presence	8.831	3.005–25.954	**<0.001**
Vascular invasion			
Absence	1		
Presence	2.563	0.509–12.907	0.254
CLNM			
Absence	1		
Presence	4.624	1.366–15.649	**0.014**
LLNM			
Absence	1		
Presence	20.243	5.193–78.917	**<0.001**

*PTMC, papillary thyroid microcarcinoma; Y, year; ETE, extrathyroidal extension; CLNM, central lymph node metastasis; LLNM, lateral lymph node metastasis; HR, Hazard ratio.95% CI, 95% confidence interval. The bold values mean the P <0.05*.

## Discussion

PTMC was found both incidentally and non-incidentally. During the past few decades, the prevalence of PTMC has increased, mainly due to the high-frequency use of US and FNA (24). As reported, the incidence of PTMC was up to 22% among benign thyroid nodules (7). In our study, the incidence of incidental PTMC was lower, at 9.7%, but the proportion of tumor size ≤ 5 mm in incidental PTMC was as high as 72.2%. There are the following reasons for the high incidence of incidental PTMC. First, US is less accurate in diagnosing thyroid lesions with diameters <5 mm, with only 53.8% accuracy (25). Second, although FNA was commonly used for preoperative diagnosis of thyroid cancer, 7.9 to 18% of the aspirates were classified as “indeterminate,” which carry the risk of malignancy (26, 27). Finally, the ATA guidelines recommend against performing FNA for thyroid lesions <1 cm as long as the lesions were confined to the thyroid (20). In fact, the cytological diagnosis is more appropriate according to the guidelines of Japan (28). Incidental PTMC patients not only faced the second operation, with associated surgical risks and additional cost, but our study also found that the recurrence rate of incidental PTMC patients was significantly higher than that of overt PTMC patients. Therefore, we suggest that detailed preoperative evaluation is essential. First, for patients with historical factors of malignancy (a history of head and neck radiation therapy, familial thyroid carcinoma, and so on), or with subnormal TSH, high-frequency ultrasonography could be performed by an experienced imaging specialist to find out the other neglected nodules. If a suspicious nodule was not found, shorter follow-up time may also helpful for early diagnosis. Second, serum TSH should be measured. If the serum TSH is subnormal, a radionuclide thyroid scan (^123^I) to assess the nodule may help to decrease the rate of incidental PTMC. For patients with isofunctioning or non-functioning nodules, FNA for nodules with highly suspicious sonographic patterns may helpful for early diagnosis. Considering the false negative rate of FNA, additional molecular analysis of thyroid tissue, including immunohistochemistry, analysis for mutations and gene rearrangements may improve the sensitivity and specificity of FNA (29, 30). Of course, microscopic evaluation of the surgical specimen still represents the gold standard for the diagnosis of thyroid nodules. Careful examination of specimens after surgery to find incidental PTMC is also very important.

The treatment of PTMC varies from observation to surgical treatment and TSH inhibition. Because of the indolent behavior of PTMC, the word “cancer” used to describe PTMC may play an integral role in the overtreatment. Some surgeons doubt whether surgery should be performed for PTMC immediately after diagnosis, or less aggressive strategies such as “watch-and-wait” may be used (31). The ATA guidelines (2015) support the active surveillance in very low-risk thyroid cancers (20). Few studies on patients' preference for active surveillance vs. surgery have been studied, and many patients were still more likely to choose surgery when cancer was detected. Moreover, we found that multifocality, tumor size >5 mm and presence of ETE, LLNM, and CLNM were significantly associated with RFS in PTMC patients. Therefore, we still suggest surgery for PTMC patients with risk factors. The most proper extent of surgery, which is based on the extent of PTMC, remains controversial. We analyzed the relationship between the extent of thyroid resection and the risk of recurrence. When PTMC patients had single focus, there was no statistical difference between TT and lobectomy in the recurrence rate, indicating that lobectomy can be regarded as appropriate for unifocal PTMC patients at present. Our result was consistent with the ATA guidelines (2015) that thyroid lobectomy alone is sufficient for unifocal PTMC patients without ETE, CLNM or familial thyroid carcinoma (20). However, in multifocal PTMC, TT significantly reduced the risk of recurrence of thyroid beds and lymph nodes. Considering that multifocality was a risk factor for RFS, we agree with the conclusion of Ito et al. that TT should be performed in patients with multifocality, otherwise lobectomy would be sufficient (32).

Although the ATA guidelines indicated a favorable prognosis for PTMC patients, with a locoregional recurrence rate of 2~6% and a metastasis rate of 1~2% (20), presence of LNM may be associated with an increased risk of local recurrence and poor survival (12, 13). With regard to lymph node dissection, we routinely prophylactically dissect the lymph nodes in the central compartment because it can be dissected without prolonging the incision. In addition, unlike reoperation after the development of recurrence, prophylactic CND can provide a clear surgical field during initial surgery. We observed that ~40.3% of the overt PTMC patients had lymph node involvement, which is consistent with the high rate of LNM (43 to 64%) of patients undergoing prophylactic CN in previous studies (16, 32). The discovery of LNM upgrades PTMC patients above 45 years old from stage I to stage III. It is worth noting that accurate staging not only helps predict the prognosis but is also critical for determining which patients need RAI treatment. Scholars who oppose prophylactic CND for cN0 patients hold the view that prophylactic CND may lead to the higher rates of postoperative complications (21). Our results confirmed previous reports that prophylactic CND could increase the rate of hypocalcemia. Temporary hypocalcemia was mainly due to transient parathyroid dysfunction caused by blood flow blocking and could be corrected by calcium supplementation after surgery. Apart from the prophylactic CND, a high proportion of TT could cause an increase of permanent hypocalcemia in patients with overt PTMC (33). In addition, we did not routinely perform intraoperative parathyroid autologous transplantation, which was another cause of the increase in permanent hypocalcemia. As for the incidence of incision infection, bleeding required resurgery, and for transient or permanent RLN injury the two groups had no statistical difference. Despite the high incidence of hypocalcemia, overt PTMC patients in our study did benefit from the CND. Compared with incidental PTMC patients, overt PTMC patients not only had a lower recurrence rate (the rate of cervical lymph nodes recurrence and thyroid bed recurrence) but also had a higher RFS. Our results were consistent with the previous study that prophylactic CND could effectively reduce the local recurrence rate and improve the prognosis (34).

Similar to previous studies (35, 36), we found that age <45 years, tumor size >5 mm and presence of ETE were independent risk factors for CLNM. The incidence of CLNM in patients with at least one risk factor was significantly higher than in patients without all risk factors. Patients with three risk factors were at highest risk of CLNM, with a rate of CLNM up to 73.9%. Since CLNM was a risk factor for RFS, we recommend that for patients with any of risk factors of CLNM, careful preoperative evaluation be required to rule out the presence of CLNM, and if CLNM is not found before surgery, routine prophylactic CND is recommended for these patients.

There are several potential limitations in our study. First, our study was a retrospective study from a single center, which might have a selection bias. Errors and biases tend to be higher in retrospective studies than in prospective studies. The data we provided were extracted from the document and were not captured in the actual conversation. Therefore, the possibility of residual confounding variables of measured or unmeasured factors could not be ruled out. Second, the follow-up time was relatively short (average period: 39 months), which may result in the low recurrence rates. Moreover, US was routinely used for detection of recurrences during the follow-up period. Due to the low sensitivity of US (25), occult metastasis may be missed. Longer follow-up period or further imaging examinations may increase the recurrence rate and make the results more stable. Third, different surgeons participated in performing thyroidectomy or lymph node dissection, and surgeon-specific factors may affect postoperative outcomes. In addition, since LND is not recommended as a preventive procedure (20), there may be undetected LLNM. Finally, our research lacked molecular and genetic analysis to further study the additional information associated with tumor aggressiveness, which reflected the nature of a retrospective study.

In conclusion, because incidental PTMC is difficult to identify with benign-appearing nodules before surgery, FNA is recommended. The combination of FNA cytologic interpretation and molecular analysis of thyroid tissue may contribute to the diagnosis of indeterminate nodules. For multifocal PTMC patients, TT should be performed to reduce the recurrence, while for unifocal PTMC patients, lobectomy is sufficient. Due to the high incidence of CLNM in PTMC patients, routine prophylactic CND can be recommended in PTMC patients with independent risk factors of CLNM. Aggressive surgery and close follow-up are essential for patients with risk factors of RFS.

## Data Availability

The datasets for this study will not be made publicly available because The database contains personal information about the patient, so our hospital does not allow public data.

## Ethics Statement

This study has been approved by the Institutional Review Board of Changzhou First People's Hospital ethics committee and has been performed according to the ethical standards laid down in the 1964 Declaration of Helsinki.

## Informed Consent

Informed consent was obtained from all individual participants included in the study.

## Author Contributions

LW took charge of conceiving and designing the study. HP was responsible for collecting the data and analyzing and interpreting the data. J-WF took charge of writing the manuscript. JY was responsible for providing critical revisions. Approving the final version of the manuscript was in charge of YJ and ZQ. All authors have contributed significantly and agree with the content of the manuscript.

### Conflict of Interest Statement

The authors declare that the research was conducted in the absence of any commercial or financial relationships that could be construed as a potential conflict of interest.

## Abbreviations

TC: thyroid cancer
PTC: papillary thyroid cancer
PTMC: papillary thyroid microcarcinoma
LNM: lymph nodes metastases
CLNM: central lymph node metastases
US: ultrasonography
CND: central neck dissection
ATA: the American thyroid association
Tg: thyroglobulin
TT: total thyroidectomy
FNA: fine-needle aspiration
LLNM: lateral lymph node metastases
LND: lateral neck dissection
ETE: extrathyroidal extension
RFS: recurrence-free survival
RLN: recurrent laryngeal nerve
TSH: thyroid-stimulating hormone
RAI: radioactive iodine.
